# A General Method for Targeted Quantitative Cross-Linking Mass Spectrometry

**DOI:** 10.1371/journal.pone.0167547

**Published:** 2016-12-20

**Authors:** Juan D. Chavez, Jimmy K. Eng, Devin K. Schweppe, Michelle Cilia, Keith Rivera, Xuefei Zhong, Xia Wu, Terrence Allen, Moshe Khurgel, Akhilesh Kumar, Athanasios Lampropoulos, Mårten Larsson, Shuvadeep Maity, Yaroslav Morozov, Wimal Pathmasiri, Mathew Perez-Neut, Coriness Pineyro-Ruiz, Elizabeth Polina, Stephanie Post, Mark Rider, Dorota Tokmina-Roszyk, Katherine Tyson, Debora Vieira Parrine Sant'Ana, James E. Bruce

**Affiliations:** 1 Department of Genome Sciences, University of Washington School of Medicine, Seattle, WA, United States of America; 2 Boyce Thompson Institute for Plant Research, Ithaca, NY, United States of America; 3 USDA-Agricultural Research Service, Ithaca, NY, United States of America; 4 Department of Plant Pathology and Plant-Microbe Biology, Cornell University, Ithaca, NY, United States of America; 5 Cold Spring Harbor Laboratory, Cold Spring Harbor, NY, United States of America; 6 Cold Spring Harbor Laboratory Proteomics Course 2016, Cold Spring Harbor, NY, United States of America; H Lee Moffitt Cancer Center and Research Institute, UNITED STATES

## Abstract

Chemical cross-linking mass spectrometry (XL-MS) provides protein structural information by identifying covalently linked proximal amino acid residues on protein surfaces. The information gained by this technique is complementary to other structural biology methods such as x-ray crystallography, NMR and cryo-electron microscopy[1]. The extension of traditional quantitative proteomics methods with chemical cross-linking can provide information on the structural dynamics of protein structures and protein complexes. The identification and quantitation of cross-linked peptides remains challenging for the general community, requiring specialized expertise ultimately limiting more widespread adoption of the technique. We describe a general method for targeted quantitative mass spectrometric analysis of cross-linked peptide pairs. We report the adaptation of the widely used, open source software package Skyline, for the analysis of quantitative XL-MS data as a means for data analysis and sharing of methods. We demonstrate the utility and robustness of the method with a cross-laboratory study and present data that is supported by and validates previously published data on quantified cross-linked peptide pairs. This advance provides an easy to use resource so that any lab with access to a LC-MS system capable of performing targeted quantitative analysis can quickly and accurately measure dynamic changes in protein structure and protein interactions.

## Introduction

Chemical cross-linking with mass spectrometry (XL-MS) has become a more widely accepted and utilized technique for the analysis of biomolecular structures [1, 2]. Information from these experiments generates distance restraints between surface exposed proximal amino acids that are reactive with the chemical cross-linker used (typically Lys). These restraints can be used to guide molecular modeling and docking experiments for protein and protein complexes. The information generated from XL-MS experiments is often complementary to other structural techniques such as X-ray crystallography, electron microscopy (EM) and nuclear magnetic resonance spectroscopy (NMR)[1]. Intermolecular cross-links provide information on protein-protein interactions, including the identity of interacting partners and regions near the interaction interface. Intramolecular cross-links provide information on the structure and conformation of proteins. As the cross-linking reaction is carried out in solution where protein structures and interactions are highly dynamic, XL-MS is able to provide a snapshot from an ensemble of protein conformations and interactions existing simultaneously.

Quantitative XL-MS (qXL-MS) extends the capabilities of XL-MS to allow for comparison of cross-linked peptides across experimental conditions and varying biological states. Importantly qXL-MS is able to provide insight on the dynamics of protein conformations, interactions and the composition of protein complexes. To date most qXL-MS studies have relied on light and heavy isotopically labeled cross-linker molecules [1, 3–5]. Recent applications of qXL-MS utilizing deuterated cross-linkers include the comparison of unphosphorylated and phosphorylated states of a spinach chloroplast ATPase [4], distinguishing conformational states of the chaperonin TRiC/CCT complex, and characterizing structural differences between the complement components C3 and C3(H2O) [5]. Extending the application of qXL-MS to a proteome scale and utilizing stable isotope labeling by amino acids in cell culture (SILAC) [6] has enabled the detection of key protein interaction and conformational changes between drug resistant and sensitive cancer cell lines [7]. qXL-MS has been shown to provide a sensitive readout for changing protein conformations and interactions induced by drug treatment of cells [8]. It was also shown that concentrations of cross-linked peptide pairs scale with drug concentration and vary depending on drug type. In that study, the targeted mass spectrometry method, parallel reaction monitoring (PRM), was used to shed light on differential conformations of Hsp90 that were enriched depending on the class of inhibitor used in the cellular treatment, N-terminal domain Hsp90 inhibitor versus C-terminal domain inhibitor. Parallel reaction monitoring involves isolation and fragmentation of selected precursor ions followed by detection of all target product ions (transitions), in one high resolution mass analysis. Processing of the PRM data required manual extraction of the chromatographic data, resulting in a time consuming and tedious process. Automation of PRM data analysis on cross-linked peptides will improve our ability to derive biological meaning and novel insights from the data.

There are limited software options for the processing of qXL-MS data. A modification to MaxQuant was made to allow for quantification of d0/d4 BS3 cross-linked peptide pairs [9]. The Aebersold lab developed xTract specifically for quantifying cross-linked peptide pairs by either stable-isotope labeling or label free quantification [10]. The majority of the methods are designed for quantification based on MS1 signal. One proof of principle study combined isobaric Tandem Mass Tags (TMT) with XL-MS to allow quantification from the reporter ion signal in MS3 spectra [11]. To date, no study has demonstrated a means for automated targeted quantification of cross-linked peptide pairs based on the specificity of MS2 signals, e.g. PRM. Targeted MS quantitative methods typically benefit from increased specificity and sensitivity, both desirable features for cross-linked peptides which are typically low abundance and produce complex fragment ion spectra. In previous work employing PRM quantification of cross-linked peptide pairs, manual extraction of the signal for the PRM transitions for specific fragment ions resulting from collision induced dissociation of PIR cross-linked peptide pairs was performed, namely the intact released peptide ions, and long arm ions which result from the cleavage of a single PIR labile bond [8].

To harness the power of automation and the sensitivity of PRM analyses, we adapted the widely used, open source software package Skyline[12] for the analysis of qXL-MS data. The end result is an easy to use resource that any lab with access to a LC-MS system capable of performing targeted quantitative analysis (such as Thermo’s QE+) can quickly probe changes to protein structures and interactions. The broad applicability of this approach is demonstrated through cross-laboratory study and comparison using the automated method described herein. One arm of the study was undertaken in the Bruce Lab using both cross-linked purified protein samples and complex biological samples from *in vivo* cross-linking on intact cultured human cells with excellent reproducibility. Students attending the 2016 Cold Spring Harbor Proteomics course utilized Skyline methods for the targeted analysis of PIR cross-linked samples from aliquots of the exact same samples. Therefore, the methods described here represent a resource to the growing structural proteomics community employing qXL-MS experiments to study biomolecular structures.

## Materials and Methods

### Preparation of PIR cross-linked BSA peptide samples

Bovine Serum Albumin (Sigma) was dissolved in 1 mL of 170 mM Na_2_HPO_4_ pH 8.0 at a concentration of 14.4 μM (1 mg/mL). PIR cross-linker (BDP-NHP) was synthesized as previously described[13], and added from a 333 mM stock solution in DMSO to a final concentration of 1 mM. The cross-linking reaction was carried out for 30 min at room temperature. After 30 min, 100 μL of 100 mM NH_4_HCO_3_ was added to quench any remaining reactive BDP-NHP. Disulfide bonds were reduced with 5 mM Tris(2-carboxyethyl)phosphine hydrochloride (TCEP, Thermo) for 30 min at room temperature and the resulting sulfhydryl groups were alkylated using 10 mM iodoacetamide (IAA, Thermo) for 30 min at room temperature. The cross-linked protein was digested using a 1:200 w/w ratio of sequencing grade modified trypsin (Promega) at 37°C for 16 h. The resulting peptide mixture was acidified to pH 2 with formic acid and desalted using C18 Sep-Pak cartridges (Waters) according to the instructions from the manufacturer. The peptide solution was then concentrated using an EZ-2 Plus vacuum centrifuge (Genevac), reconstituted with 0.1% formic acid at a concentration of 1 mg/mL, and stored at -80°C until LC-MS/MS analysis.

### Preparation of cross-linked SILAC labeled cell samples

Cross-linked SILAC labeled cell samples were prepared as described previously [8]. Briefly, HeLa cells (ATCC) were seeded at a density of 5 x 10^6^ cells into 150 mm culture dishes with 20 mL of SILAC DMEM medium containing either isotopically light L-lysine and L-arginine or isotopically heavy ^13^C_6_^15^N_2_-L-lysine and ^13^C_6_-L-arginine (Silantes) and supplemented with 10% dialyzed FBS (Valley Biomedical Inc.) and 1% pen./strep. (Fisher Scientific). Cells were treated with various concentrations (100, 250, 500, 1000 nM) Tanespimycin (17-N-allylamino-17-demethoxygeldanamycin [17-AAG] Cayman Chemicals or 0.1% v/v dimethyl sulfoxide (DMSO) for 18 h before harvesting with 5 mL of phosphate buffered saline (PBS) containing 5 mM EDTA. Cells were washed with PBS and pelleted by centrifugation at 300 x g for 3 min and suspended in 0.3 mL of 170 mM Na_2_HPO_4_ pH 8.0 for chemical cross-linking.

Light and heavy isotopically-labeled cells were mixed at equal numbers (2 x 10^7^ cells), either light 17-AAG treated/ heavy control or light control/ heavy 17-AAG treated, before adding the PIR cross-linker (BDP-NHP) from a concentrated stock solution (333 mM) in DMSO to a final concentration of 10 mM. The reaction was carried out at room temperature for 1 h with constant mixing. The reaction time of 1 h allows time for over 99% of the cross-linker to react based on the half-life of 7.5 min measured by monitoring the absorbance of released N-hydroxyphthalamide, see S1 Fig. After 1 h the cells were pelleted by centrifugation at 300 g for 3 min the supernatant was removed and the cross-linking reaction was quenched by suspending the cell pellet in 0.1 M NH_4_HCO_3_ pH 8.0.

Cross-linked cells were lysed by suspending the cell pellet in ice cold 8 M urea solution in 0.1 M tris buffer pH 8.0. Sample was sonicated using a GE– 130 ultrasonic processor, followed by reduction and alkylation and tryptic digestion. Resulting peptides were fractionated by strong cation exchange chromatography (SCX) using an Agilent 1200 series HPLC system equipped with a 250 x 10.0 mm column packed with Luna 5 μm 100 Å particles (Phenomenex). Cross-linked peptides from SCX fractions were further enriched using UltraLink monomeric avidin (Thermo). The enriched cross-linked peptide sample was concentrated by vacuum centrifugation and stored at -80°C until LC-MS analysis.

### LC-MS/MS analysis performed in the Bruce Lab

Reversed phase chromatographic separation of cross-linked peptide samples was performed using an EASY-nLC 1000 system (Thermo Scientific) equipped with a 3 cm x 100 um trapping column and a 60 cm x 75 um analytical column both packed with 5 um Reprosil C8 particles with 120 angstrom pores (Dr. Maisch GmbH). Peptides were loaded onto the trapping column using 20 uL of solvent A (H_2_0 containing 0.1% formic acid) at a flow rate 2 uL/min. Reversed phase separation over the analytical column was performed by applying a linear gradient from 98% solvent A and 2% solvent B (acetonitrile containing 0.1% formic acid) to 60% solvent A and 40% solvent B over 120 minutes at a flow rate of 300 nL/min. Eluting peptides were ionized by electrospray ionization by applying a voltage of 2.2 kV to a laser pulled tip at the tip of the analytical column. Mass spectrometry was performed using Q-Exactive Plus mass spectrometer (Thermo Scientific) operated using a PRM method with the following settings: a resolving power of 17,500 @ 200 *m/z*, automatic gain control (AGC) target of 2E5 ions, maximum ion time of 100 ms, isolation window of 3 *m/z* and a normalized collision energy of 27.

### LC-MS/MS analysis performed at Cold Spring Harbor Laboratories

Reversed phase chromatographic separation of cross-linked peptide samples was performed using an EASY-nLC 1200 system coupled with a Q-Exactive High Field mass spectrometer (Thermo Scientific). The nano-flow LC system was configured with a 20 mm, 75 micron ID PepMap100 C18 trap column (Thermo Scientific), and a 15 cm, 50 micron ID PepMap RSLC C18 analytical column (Thermo Scientific) with an steel emitter. Mobile phase A consisted of 2% acetonitrile/0.1% formic acid and mobile phase B consisted of 80% acetonitrile/ 0.1% formic acid. Samples containing cross-linked peptide pairs were injected through the autosampler onto the trap column. Peptides were then separated using the following linear gradient steps at a flow rate of 300 nL/min: 2% B for 1 min, 2% B to 35% B over 100 min, 35% B to 40% B over 5 min, 40% B to 80% B over 1 min, held at 80% B for 4 min, 80% B to 2% B over 1 min and held at 2% B for 8 min. Separation was accomplished with a 40 min linear gradient from 2% to 40% solvent B at 300 nL/min. The mass spectrometer was operated with a PRM method with the following settings: a resolving power of 15,000 @ 200 *m/z*, ACG target of 2E5 ions, maximum ion time of 10 ms, isolation window of 1 *m/z* and a normalized collision energy of 27.

### ReACT2prm

To generate a transition table file for importing the cross-linked peptide information into Skyline, the Linux command line executable program, ReACT2prm, was developed in C. The program has several options for the precursor ion *m/z* values to use. The default option is to use the *m/z* value for the most abundant isotope for a given cross-linked peptide pair based on the calculation of the theoretical isotope distribution[14]. The other options for the precursor *m/z* are to use the experimentally observed *m/z* from the ReACT analysis that identified the cross-linked peptide pair, or the theoretical monoisotopic *m/z* value. An additional option to change the mass of the reporter ion of the PIR cross-linker exists for support of multiple PIR chemical cross-linker compounds. The program calculates the *m/z* values for the theoretical fragment ions including the full length released peptides, long arm ions resulting from the cleavage of a single PIR labile bond, and the y-type and b-type ions from both cross-linked peptides assuming both PIR labile bonds were cleaved. ReACT2prm creates a tab-delimited text file suitable for importing into Skyline for PRM analysis. ReACT2prm and the source code is available for download at http://brucelab.gs.washington.edu/software.html.

### Importing transition lists and data into Skyline

Skyline (v. 3.5.0.9319) was used to build targeted ion isolation lists as well as import and analyze PRM data from PIR cross-linked peptides. The text output from the ReACT2prm program was inserted as a Skyline transition list with the following settings: Small molecules transition list with columns in this order: precursor name, precursor *m/z*, product *m/z*, precursor charge, product charge, molecule list name, product name. After insertion of the transition list data the check for errors function is run. If this passes the insert button is pressed to load the transitions into Skyline. At this point an isolation list file can be exported from Skyline for use in constructing a PRM method for operation of the mass spectrometer. After insertion of the transition list the file needs to be saved before importing the RAW mass spectrometry data files. A tutorial for utilizing Skyline for cross-linked peptide pair is described (**S1 Text**)

## Results and Discussion

### General Experimental Approach

**Fig 1:**
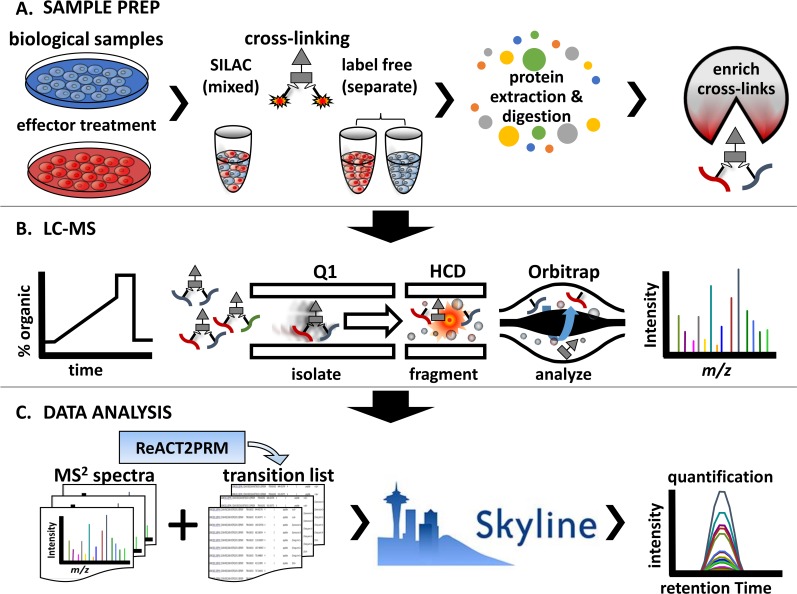
Experimental outline. **A.** Biological samples are prepared for qXL-MS comparing two or more conditions. The samples are treated with chemical cross-linker either as (1) a mixed sample if SILAC labeling was used or (2) as separate samples if carrying out a label free experiment or using isotopically labeled cross-linkers. Following the cross-linking reaction proteins are extracted, enzymatically digested, and subjected to various strategies (i.e. strong cation exchange and affinity chromatography) for enrichment cross-linked peptide pairs. **B.** LC-MS analysis of samples enriched for cross-linked peptide pairs is carried out. This consists of reversed phase chromatographic separation by LC followed by analysis by MS. The mass spectrometer is operated in PRM mode where an inclusion list of *m/z* values for the precursor ions of interest is used to target specific cross-linked peptides. The PRM mass spectrometric analysis used here consists of three steps including isolation of precursor ions, fragmentation by collision with neutral gasses, and detection of mass to charge ratios of the resulting fragment ions. **C)** Resulting MS2 data are converted into transition lists and imported into Skyline for analysis.

The general approach for performing large scale targeted quantitative cross-linking studies is outlined in **Fig 1**. Two or more biological samples which differ in biological state due phenotypic differences or due to effector treatment are generated **Fig 1A**. Samples enriched for cross-linked peptide pairs are analyzed by LC-MS (**Fig 1B**). The resulting mass spectrometric data are then converted into theoretical transition lists and processed using Skyline for quantification as described in more detail below (**Fig 1C**).

### Adaptation of Skyline for PRM data from PIR cross-linked peptides

Skyline is a software package frequently used for building and optimization of targeted proteomics methods (SRM, PRM, DIA) and quantitative analysis of the resulting mass spectrometric data [12]. Skyline predicts peptide fragment ion masses based on peptide sequences resulting from *in silico* digestion of protein sequences. To date, there are no publications describing the use of Skyline for analysis of cross-linked peptides. Recent advances in PIR and other cross-linking experiments have resulted in the reliable identification of hundreds to thousands of cross-linked peptide pairs, each providing information on the conformation of protein and protein complex structures [3, 7, 8, 13, 15–18]. Furthermore, quantitative principles from traditional proteomics experiments have been extended to cross-linking experiments allowing for insight into the dynamic changes of protein conformations and interactions [7]. Software for the analysis of resulting data from such experiments is lacking. Therefore, we adapted the features of Skyline for the analysis of data from quantitative PIR cross-linking experiments. To facilitate this, the software tool ReACT2prm was written to calculate the fragment ions for PIR cross-linked peptides including the singly charged y and b type ions from both peptides as well as the ions generated from cleavage of the engineered MS labile bonds in the PIR cross-linker. See **S2 Fig** for further explanation of the fragment ions considered. Importantly ReACT2prm calculates the *m/z* values for the transitions based on the peptide sequences output from a Comet[19] search, which can include masses for post-translational and chemical modifications to peptides. This allows for inclusion of residual PIR mass modification or so-called “stump mass” as well as any isotope labels resulting from SILAC or isotope labeled cross-linker molecules as demonstrated below. The resulting table in the form of a tab-delimited text file can input into Skyline as a transition list using the small molecule setting. The following information is input into the transition list: precursor name, precursor ion *m/z*, product ion *m/z*, precursor charge, product charge, molecule list name, product name. An example of an input file is included as **S2 Text.** Skyline documents containing the PRM data can be easily shared between researchers in different laboratories using Panorama, an online application for storing, sharing, analyzing, and reusing targeted assays created and refined with Skyline [20]

### Label free quantification of BSA cross-linked peptide pairs

**Fig 2:**
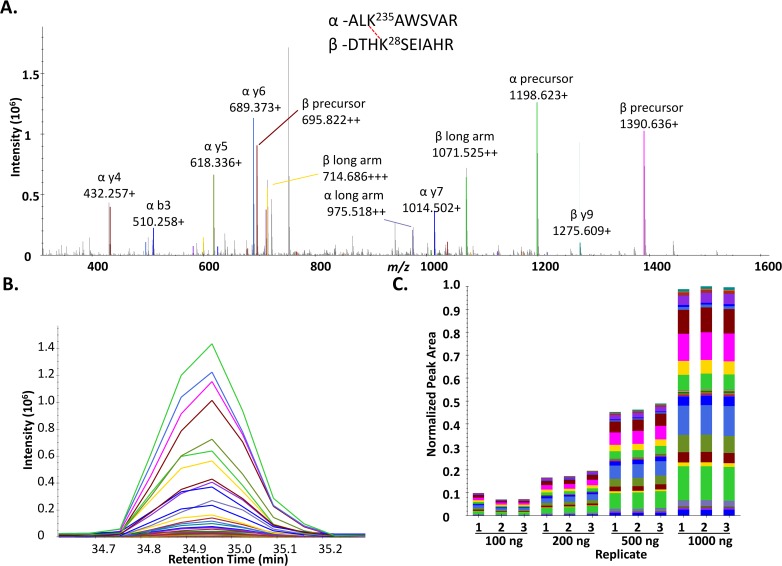
Quantification of BSA cross-linked peptide pairs with Skyline. **A.** MS2 spectrum for the cross-linked peptide pair linking residues K235-K28 (ALK^235^AWSVAR_DTHK^28^SEIAHR), obtained from a 500 ng injection of cross-linked BSA digest. **B.** Extracted ion chromatograms for the PRM transitions observed for the cross-linked peptide pair in A. **C.** Skyline generated bar plot illustrating the normalized peak areas for the cross-linked peptide pair linking K28-K235. Peak areas are shown for triplicate analyses of varying injection amounts (100, 200, 500, and 1000 ng cross-linked BSA digest). Bars are color coded to indicate the contribution of each individual transition to the total peak area and match the color scheme in panel B.

As an initial demonstration of the use of Skyline for quantification of cross-linked peptide pairs, a set of 30 cross-linked peptide pairs (**S1 Table**) resulting from PIR cross-linking of BSA was analyzed by label free quantitative PRM analysis on a Q-Exactive Plus mass spectrometer. The 25 non-redundant residue to residue linkages identified in the 30 cross-linked peptide pairs display excellent agreement when mapped to a crystal structure of BSA (PDB 3V03) **S3 Fig**. Varying amounts of BDP cross-linked BSA digest were injected (100, 200, 500, 1000 ng) and analyzed by LC-MS. The resulting raw files were then imported into Skyline for quantitative analysis of the data. A representative MS2 spectrum of a cross-linked peptide pair from BSA linking residues K235-K28 (ALK^235^AWSVAR-DTHK^28^SEIAHR) as analyzed by Skyline is shown in **Fig 2A**. The peaks in the spectrum are color coded according to the type of fragment ion they match, listed in the box above the spectrum. A PRM extracted ion chromatographic peak for this cross-linked peptide pair is shown in **Fig 2B**. Each of the colored traces represent a PRM transition or signal from a fragment ion from the cross-linked peptide pair color coded to match the spectrum shown in **Fig 2A**. As can be seen in **Fig 2B** the most abundant transition signal results from the singly charged fragment ion resulting from cleavage of the labile D-P bond in the PIR cross-linker releasing the intact α-peptide (ALK^235^AWSVAR) detected at *m/z* 1198.6233. The released, intact β-peptide (DTHK^28^SEIAHR) was the third most abundant PRM transition, detected as a singly charged ion at *m/z* 1390.6364. Observation of ions resulting from specific cleavage of the labile PIR bonds helps to confirm the identity of the cross-linked peptide pair and is useful in assigning the correct peak in Skyline. However, in addition to these PIR specific ion signals, signals from y-type and b-type fragment ions resulting from cleavage of the backbone peptide bonds from the α-peptide and β-peptide are also detected and can be used for quantifying the cross-linked peptide pair. **Fig 2C** illustrates the quantification of the cross-linked peptide pair linking K28-K235 across an order of magnitude in concentrations resulting from triplicate analyses of injections containing 100, 200, 500 and 1000 nanograms of cross-linked BSA digest. Excellent reproducibility across replicate injections was observed and as expected the normalized peak area increases linearly with increasing injection amount.

### Cross-laboratory study demonstrates robust applicability of method

**Fig 3:**
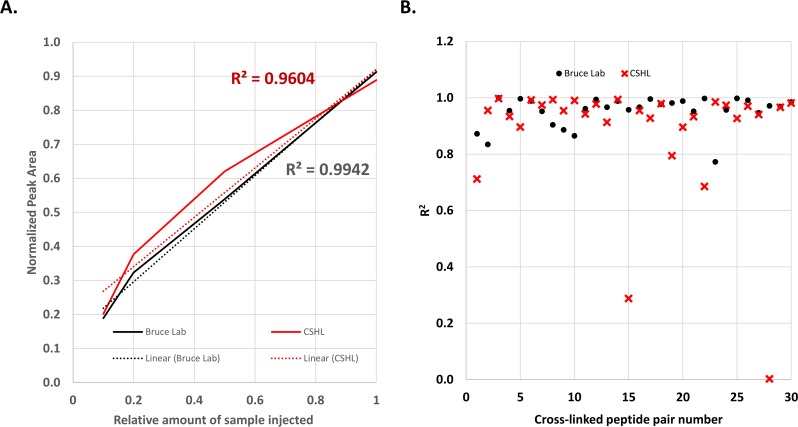
Cross-laboratory quantification of BSA-BDP cross-linked peptide pairs. **A.** Average cross-linked peptide response curve comparing data collected in the Bruce Lab and CSHL for 30 cross-linked peptide pairs. **B.** Scatter plot illustrating the R^2^ values for linear regression analysis of the data shown in A and B.

PRM and MRM methods currently form the basis for robust, widely applied quantitative measurements of peptides in proteome samples [21, 22]. In principle, PRM methods should also enable targeted quantitative analysis of cross-linked peptide pairs to greatly increase utility for protein conformational and interaction studies. To investigate this possibility, Skyline documents containing PRM methods and PIR cross-linked samples were provided to students attending the 2016 Proteomics Course at Cold Spring Harbor Laboratory (CSHL). Using the supplied PRM transitions the students repeated the analysis of the BSA cross-linked peptide pairs. As shown in **Fig 3** excellent agreement was observed between the results obtained from the analysis carried out at CSHL and the Bruce Lab. A plot displaying the concentration dependent signal for the 30 BSA cross-linked peptide pairs collected in the Bruce Lab is shown in **S4 Fig**. A plot displaying concentration dependent signal for the 30 BSA cross-linked peptide pairs collected at CSHL is shown in **S5 Fig**. In data collected from both labs, excellent linearity was observed for the signal from the 30 targeted cross-linked peptide pairs across the concentration range as demonstrated by the average linear response curves shown in **Fig 3A**. The distribution of the square of the Pearson product-moment correlation coefficients (R^2^) values is shown in **Fig 3B,** where the median value is 0.97. While the majority of cross-linked peptide pairs displayed linear trends in both sets of data, two examples were observed with relatively low R^2^ values in the CSHL data. The cross-linked peptide pair linking K228 to K374 (CASIQK^228^FGER_LAK^374^EYEATLEECCAK) had an R^2^ value of 0.29, and the cross-linked peptide pair linking K445 to K548 (SLGK^445^VGTR_K^548^QTALVELLK) had an R^2^ value of 0.0025. Both of these examples display attenuated signal at higher concentration levels resulting in a deviation from the expected linear trend, and elute at similar times in the chromatogram, 26.5 and 26.8 min respectively. A potential reason for this observation is chromatographic effects due to the shorter LC gradient length in the CSHL analyses. As evidenced by the data collected in the Bruce Lab, running a longer LC gradient resolves this issue. The Skyline documents containing the data for the cross-linked BSA peptides can be obtained from https://panoramaweb.org/labkey/PRM_XLMS.url and https://figshare.com/articles/Skyline_documents_for_the_article_A_general_method_for_targeted_quantitative_cross-linking_mass_spectrometry_/4240073. Together these results demonstrate the principle of utilizing Skyline for quantification of cross-linked peptide pairs from a purified protein under idealized conditions.

**Fig 4:**
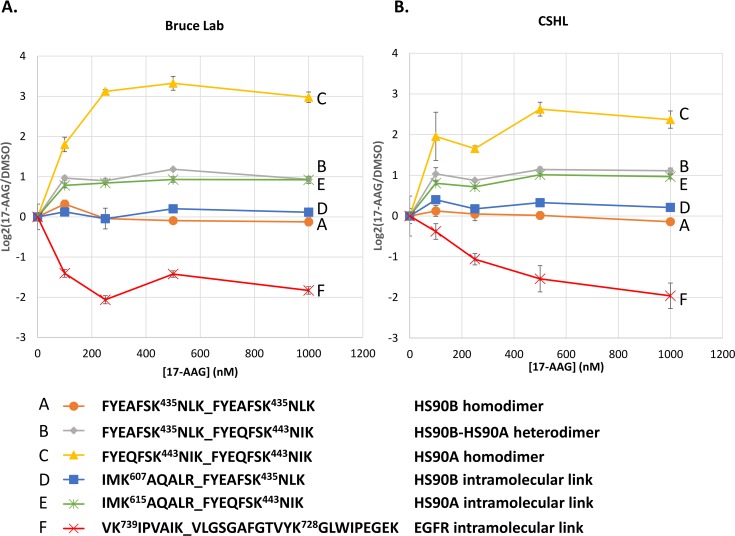
Cross-laboratory quantification of Hsp90 cross-linked peptide pairs. **A.** Plot of relative levels of cross-linked peptide pairs versus concentration of 17-AAG as quantified by PRM and Skyline in the Bruce Lab. Ratios were normalized to zero for the no drug control sample.**B.** Plot of relative levels of cross-linked peptide pairs versus concentration of 17-AAG as quantified by PRM and Skyline at CSHL.

Next, the use of PRM and Skyline for quantifying cross-linked peptide pairs resulting from large scale *in vivo* cross-linking experiments was demonstrated. For this purpose, several cross-linked peptide pairs were selected for targeted PRM analysis that were identified in a previous study employing SILAC labeling and chemical cross-linking to investigate the effect of 17-AAG treatment in cells [8]. 17-AAG is an inhibitor of Hsp90 which binds to the ATP binding pocket in the N-terminal domain (NTD) of Hsp90 effectively shutting down its chaperone function [23, 24]. In the previous study, quantitative changes to cross-linked peptide pair levels were observed that indicated 17-AAG concentration dependent conformational and interaction changes to Hsp90 and its interaction partners [8]. As with the cross-linked BSA samples discussed above, PRM analysis of these targeted cross-linked peptide pairs was carried out in the Bruce Lab and at CSHL. For this analysis each cross-linked peptide pair exists as a light and heavy isotope version which are independently targeted for PRM analysis. Relative ratios of the light and heavy isotopic partners can then be calculated from the total PRM transition chromatographic peak areas. Excellent reproducibility was observed between the data collected in the two laboratories for the concentration dependent response for a number of cross-linked peptide pairs (**Fig 4**). Importantly these data also agree with the MS1 based quantification and PRM data which was analyzed by manual extraction of the transition chromatographic peaks in our previous study [8]. As can be seen in the colored traces (**Fig 4A and 4B**) the relative levels of the targeted cross-linked peptide pairs displayed differing trends with increasing 17-AAG treatment of cells. The orange (A) and blue (D) traces stay relatively constant across the 17-AAG concentration. These traces represent cross-linked peptide pairs (homodimer: FYEAFSK^435^NLK-FYEAFSK^435^NLK, orange trace (A); and intramolecular link: IMK^607^AQALR-FYEAFSK^435^NLK, blue trace (D)) from the beta isoform of Hsp90 (HS90B), which is constitutively expressed and not expected to change with inhibitor treatment. The yellow traces (C) represent a cross-linked peptide pair (FYEQFSK^443^NIK- FYEQFSK^443^NIK) identifying a homodimer of the alpha isoform of Hsp90 (HS90A), which is inducible under conditions of stress including treatment with Hsp90 inhibitors which bind the NTD. A strong increase in the relative levels of this cross-linked peptide pair were observed with 17-AAG treatment of cells in the data collected in the Bruce Lab as well as CSHL. The green trace (E) represents the HS90A intramolecular link (IMK^615^AQALR- FYEQFSK^443^NIK homologous to the blue trace (D) for HS90B, and displays increasing levels with 17-AAG treatment in contrast to the homologous HS90B link (607–435, blue trace (D)). Similarly, the grey trace (B) represents a the HS90B-HS90A heterodimer (FYEAFSK^435^NLK- FYEQFSK^443^NIK) which was observed with increasing levels with 17-AAG treatment. Lastly the red trace (F) represents an intramolecular cross-link (VK^739^IPVAIK-VLGSGAFGTVYK^728^GLWIPEGEK) in the Hsp90 client protein EGFR displays decreasing levels with 17-AAG treatment, as would be expected since EGFR stability depends on Hsp90 chaperone function. The Skyline documents containing the data for the cross-linked peptides from 17-AAG treated SILAC cells can be obtained from https://panoramaweb.org/labkey/PRM_XLMS.url and https://figshare.com/articles/Skyline_documents_for_the_article_A_general_method_for_targeted_quantitative_cross-linking_mass_spectrometry_/4240073. Together these data illustrate that previously identified cross-linked peptide pairs from large scale in vivo cross-linking efforts can be reliably detected and quantified using the PRM methods described herein. This makes large repositories of cross-linked peptide pairs, such as XlinkDB [25, 26], increasingly important as now any of the cross-linked peptide pairs existing therein can serve as readily available targets for researchers to probe cross-linked peptide pairs representing a particular conformation of a protein or interactions between proteins with varying biological stimuli. This is analogous to the SRMAtlas [27] for tracking protein expression levels in standard quantitative proteomics experiments, only now providing molecular structure and interaction information. To facilitate the generation of targeted assays for cross-linked peptide pairs, new functionality has been added to XlinkDB allowing users to generate a PRM transition list for any cross-linked peptide pair including those present in the database (http://xlinkdb.gs.washington.edu/xlinkdb/prmTransitionForm.php) (**S6 Fig)**. Our hope is that these types of measurements will serve as a new tool and will be adopted by the wider biological community to better understand the critical role of protein structures and interactions in driving biological function.

## Conclusions

The ever expanding application of XL-MS experiments necessitates the need for easy to use methods and software. Here the general methodology for carrying out targeted quantitative LC-MS analysis of cross-linked peptide pairs is presented. Primarily, adapting the use of Skyline, a freely available, open source software platform which has already been widely adopted by the proteomics community, for the analysis of PIR cross-linked peptides allows for a mechanism to share isolation methods and quantitative results between laboratories. This concept was demonstrated by performing label free quantitative analysis of a standard cross-linked protein digest as well utilizing isotope labeling quantitative analysis of cross-linked peptides enriched from SILAC labeled cultured human cells in two separate laboratories. Data collected between the two laboratories, one specializing in the development of chemical cross-linking mass spectrometry technology and the other focused on teaching fundamentals of proteomics research to scientist with a wide variety of backgrounds and specialties, were in excellent agreement, supporting the general applicability of developed PRM methods for protein conformational and interaction studies. Therefore, development of cross-linked peptide pair databases and associated PRM methods can greatly extend such studies to many laboratories. The vast majority of students in the course are biologists who are interested to collaborate with proteomics researchers and core facilities to apply advanced methods in their biological systems. The study also clearly demonstrates that with hands-on training in data collection and analysis, very little now stands in the way of biologists to successfully apply highly technical approaches for quantitative protein interaction studies. Therefore, development of cross-linked peptide pair databases and associated PRM methods can greatly extend such studies to many laboratories who are currently limited to using molecular and genetic approaches to study protein interactions.

## Supporting Information

S1 Fig Half-life of BDP-NHP cross-linker. Plot of the absorbance at 410 nm of N-hydroxylphalamide as it is released during the hydrolysis reaction of 1 mM BDP-NHP cross-linker in a solution of 170 mM Na2HPO4, pH 8.0. The measured half-life is approximately 7.5 min.
(PDF)

S2 Fig Fragment ions generated from PIR cross-linked peptide pairs. **A.** Schematic illustrating the PIR specific fragment ions generated upon collision induced dissociation of a cross-linked peptide pair linking K235 and K28 of BSA. Dissociation of the MS labile, aspartyl-prolyl peptide bonds in the PIR molecule result in the formation of released intact peptide ions for both the alpha and beta peptides which contain a residual mass modification (197.032 Da) referred to as a “stump mass” on the side chain of the cross-linked Lys. Dissociation of a single MS labile bond also results in the formation fragment ions containing a residual mass modification of (948.444 Da) on either the alpha and or beta peptides, referred to “long arm” ions. The reporter ion generated from dissociation of both MS labile bonds is observed at 752.412 m/z but is not generally used as a transition in PRM due to the non-specific nature of it being formed from dissociation of all PIR containing ions. **B.** MS2 spectrum for the cross-linked peptide pair mentioned in A with major fragment ions annotated.
(PDF)

S3 Fig Cross-linked sites mapped onto BSA crystal structure. Crystal structure of BSA (PDB 3V03) displayed as a ribbon structure with cross-linked Lys sites shown as green space filled models. The distances, in angstroms, between alpha carbon atoms of cross-linked Lys residues are shown as black labels. Note the residue numbering of PDB 3V03 differs from the BSA sequence numbering (UniProt entry ALBU_BOVIN) by -24 (i.e. K4 on structure is K28 in sequence). NGL viewer was used to create this image[28].
(PDF)

S4 Fig Bruce Lab PRM analysis of 30 BSA cross-linked peptide pairs. (PDF)

S5 Fig CSHL PRM analysis of 30 BSA cross-linked peptide pairs. (PDF)

S6 Fig Example page of generation of PRM transitions for a cross-linked peptide pair from XlinkDB. Entry form for PRM transition calculator can be obtained at the following URL: http://xlinkdb.gs.washington.edu/xlinkdb/prmTransitionForm.php.
(PDF)

S1 Table 30 BSA cross-linked peptide pairs selected for PRM. (XLSX)

S1 Text Protocol for using Skyline for cross-linked peptides. (PDF)

S2 Text Input PRM transition list for 30 BSA cross-linked peptides. (TXT)

## References

[1] Leitner A, Faini M, Stengel F, Aebersold R. Crosslinking and Mass Spectrometry: An Integrated Technology to Understand the Structure and Function of Molecular Machines. Trends Biochem Sci. 2016;41(1):20–32. 10.1016/j.tibs.2015.10.008 26654279

[2] Holding AN. XL-MS: Protein cross-linking coupled with mass spectrometry. Methods. 2015;89:54–63. 10.1016/j.ymeth.2015.06.010 26079926

[3] Tan D, Li Q, Zhang MJ, Liu C, Ma C, Zhang P, et al Trifunctional cross-linker for mapping protein-protein interaction networks and comparing protein conformational states. Elife. 2016;5. PubMed Central PMCID: PMCPMC4811778. 10.7554/eLife.12509 PMC4811778 26952210

[4] Schmidt C, Zhou M, Marriott H, Morgner N, Politis A, Robinson CV. Comparative cross-linking and mass spectrometry of an intact F-type ATPase suggest a role for phosphorylation. Nat Commun. 2013;4:1985 PubMed Central PMCID: PMCPMC3709506. 10.1038/ncomms2985 23756419 PMC3709506

[5] Chen ZA, Pellarin R, Fischer L, Sali A, Nilges M, Barlow PN, et al Structure of complement C3(H2O) revealed by quantitative cross-linking/mass spectrometry and modelling. Mol Cell Proteomics. 2016. 10.1074/mcp.M115.056473 PMC4974347 27250206

[6] Ong SE, Mann M. A practical recipe for stable isotope labeling by amino acids in cell culture (SILAC). Nat Protoc. 2006;1(6):2650–60. 10.1038/nprot.2006.427 17406521

[7] Wu X, Held K, Zheng C, Staudinger BJ, Chavez JD, Weisbrod CR, et al Dynamic Proteome Response of Pseudomonas aeruginosa to Tobramycin Antibiotic Treatment. Mol Cell Proteomics. 2015;14(8):2126–37. PubMed Central PMCID: PMCPMC4528242. 10.1074/mcp.M115.050161 26018413 PMC4528242

[8] Chavez JD, Schweppe DK, Eng JK, Bruce JE. In Vivo Conformational Dynamics of Hsp90 and Its Interactors. Cell Chem Biol. 2016;23(6):716–26. 10.1016/j.chembiol.2016.05.012 27341434 PMC5012217

[9] Chen ZA, Fischer L, Cox J, Rappsilber J. Quantitative cross-linking/mass spectrometry using isotope-labeled cross-linkers and MaxQuant. Mol Cell Proteomics. 2016. 10.1074/mcp.M115.056481 PMC4974350 27302889

[10] Walzthoeni T, Joachimiak LA, Rosenberger G, Rost HL, Malmstrom L, Leitner A, et al xTract: software for characterizing conformational changes of protein complexes by quantitative cross-linking mass spectrometry. Nat Methods. 2015;12(12):1185–90. 10.1038/nmeth.3631 26501516 PMC4927332

[11] Yu C, Huszagh AS, Viner R, Novitsky EJ, Rychnovsky SD, Huang L. Developing a Multiplexed Quantitative Cross-linking Mass Spectrometry Platform for Comparative Structural Analysis of Protein Complexes. Anal Chem. 2016. 10.1021/acs.analchem.6b03148 PMC5361889 27626298

[12] MacLean B, Tomazela DM, Shulman N, Chambers M, Finney GL, Frewen B, et al Skyline: an open source document editor for creating and analyzing targeted proteomics experiments. Bioinformatics. 2010;26(7):966–8. PubMed Central PMCID: PMCPMC2844992. 10.1093/bioinformatics/btq054 20147306 PMC2844992

[13] Weisbrod CR, Chavez JD, Eng JK, Yang L, Zheng C, Bruce JE. In vivo protein interaction network identified with a novel real-time cross-linked peptide identification strategy. J Proteome Res. 2013;12(4):1569–79. PubMed Central PMCID: PMCPMC3925062. 10.1021/pr3011638 23413883 PMC3925062

[14] Yergey JA. A general approach to calculating isotopic distributions for mass spectrometry. Int J Mass Spectrom Ion Phys. 1983;52(2):337–49. 10.1002/jms.4498 31957110

[15] Chavez JD, Weisbrod CR, Zheng C, Eng JK, Bruce JE. Protein interactions, post-translational modifications and topologies in human cells. Mol Cell Proteomics. 2013;12(5):1451–67. PubMed Central PMCID: PMCPMC3650351. 10.1074/mcp.M112.024497 23354917 PMC3650351

[16] Schweppe DK, Harding C, Chavez JD, Wu X, Ramage E, Singh PK, et al Host-Microbe Protein Interactions during Bacterial Infection. Chem Biol. 2015;22(11):1521–30. 10.1016/j.chembiol.2015.09.015 26548613 PMC4756654

[17] Liu F, Rijkers DT, Post H, Heck AJ. Proteome-wide profiling of protein assemblies by cross-linking mass spectrometry. Nat Methods. 2015;12(12):1179–84. 10.1038/nmeth.3603 26414014

[18] Kaake RM, Wang X, Burke A, Yu C, Kandur W, Yang Y, et al A new in vivo cross-linking mass spectrometry platform to define protein-protein interactions in living cells. Mol Cell Proteomics. 2014;13(12):3533–43. PubMed Central PMCID: PMCPMC4256503. 10.1074/mcp.M114.042630 25253489 PMC4256503

[19] Eng JK, Jahan TA, Hoopmann MR. Comet: an open-source MS/MS sequence database search tool. Proteomics. 2013;13(1):22–4. 10.1002/pmic.201200439 23148064

[20] Sharma V, Eckels J, Taylor GK, Shulman NJ, Stergachis AB, Joyner SA, et al Panorama: a targeted proteomics knowledge base. J Proteome Res. 2014;13(9):4205–10. PubMed Central PMCID: PMCPMC4156235. 10.1021/pr5006636 25102069 PMC4156235

[21] Picotti P, Aebersold R. Selected reaction monitoring-based proteomics: workflows, potential, pitfalls and future directions. Nat Methods. 2012;9(6):555–66. 10.1038/nmeth.2015 22669653

[22] Bourmaud A, Gallien S, Domon B. Parallel reaction monitoring using quadrupole-Orbitrap mass spectrometer: Principle and applications. Proteomics. 2016;16(15–16):2146–59. 10.1002/pmic.201500543 27145088

[23] Schulte TW, Neckers LM. The benzoquinone ansamycin 17-allylamino-17-demethoxygeldanamycin binds to HSP90 and shares important biologic activities with geldanamycin. Cancer Chemother Pharmacol. 1998;42(4):273–9. 10.1007/s002800050817 9744771

[24] Trepel J, Mollapour M, Giaccone G, Neckers L. Targeting the dynamic HSP90 complex in cancer. Nat Rev Cancer. 2010;10(8):537–49. 10.1038/nrc2887 20651736 PMC6778733

[25] Zheng C, Weisbrod CR, Chavez JD, Eng JK, Sharma V, Wu X, et al XLink-DB: database and software tools for storing and visualizing protein interaction topology data. J Proteome Res. 2013;12(4):1989–95. PubMed Central PMCID: PMCPMC3744611. 10.1021/pr301162j 23413830 PMC3744611

[26] Schweppe DK, Zheng C, Chavez JD, Navare AT, Wu X, Eng JK, et al XLinkDB 2.0: integrated, large-scale structural analysis of protein crosslinking data. Bioinformatics. 2016;32(17):2716–8. 10.1093/bioinformatics/btw232 27153666 PMC5013903

[27] Kusebauch U, Campbell DS, Deutsch EW, Chu CS, Spicer DA, Brusniak MY, et al Human SRMAtlas: A Resource of Targeted Assays to Quantify the Complete Human Proteome. Cell. 2016;166(3):766–78. 10.1016/j.cell.2016.06.041 27453469 PMC5245710

[28] Rose AS, Hildebrand PW. NGL Viewer: a web application for molecular visualization. Nucleic Acids Res. 2015;43(W1):W576–9. PubMed Central PMCID: PMCPMC4489237. 10.1093/nar/gkv402 25925569 PMC4489237

