# Supplementary figures and images for: A General Method for Targeted Quantitative Cross-Linking Mass Spectrometry
Source: PLoS One. 2016 Dec 20;11(12):e0167547. doi: 10.1371/journal.pone.0167547 (PMC5172568; doi:10.1371/journal.pone.0167547)

# 1mM BDP-NHP Hydrolysis (Absorbance @ 410 nm)

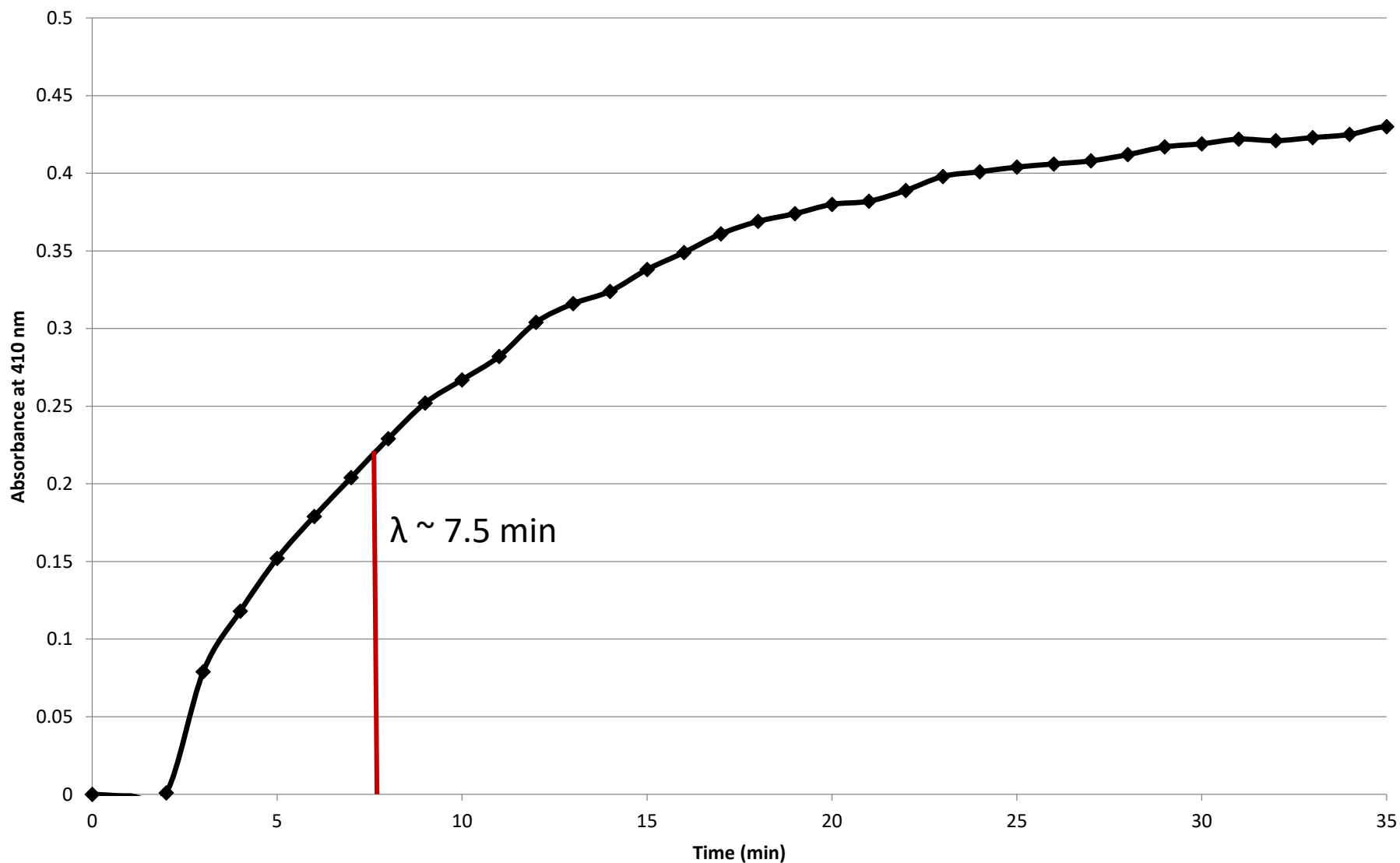

Supplement: S1 Fig — Plot of the absorbance at 410 nm of N-hydroxylphalamide as it is released during the hydrolysis reaction of 1 mM BDP-NHP cross-linker in a solution of 170 mM Na2HPO4, pH 8.0. The measured half-life is approximately 7.5 min. (PDF) [file pone.0167547.s001.pdf]

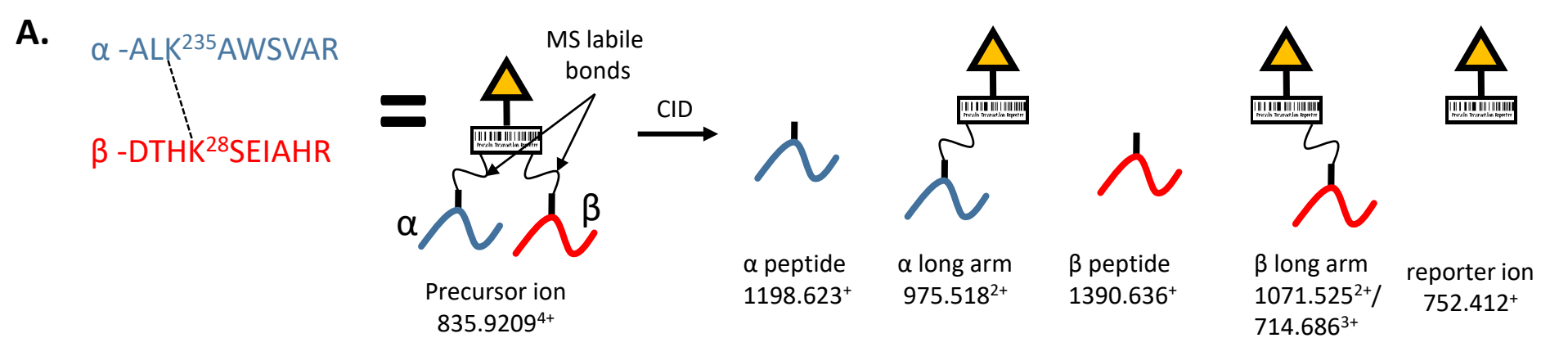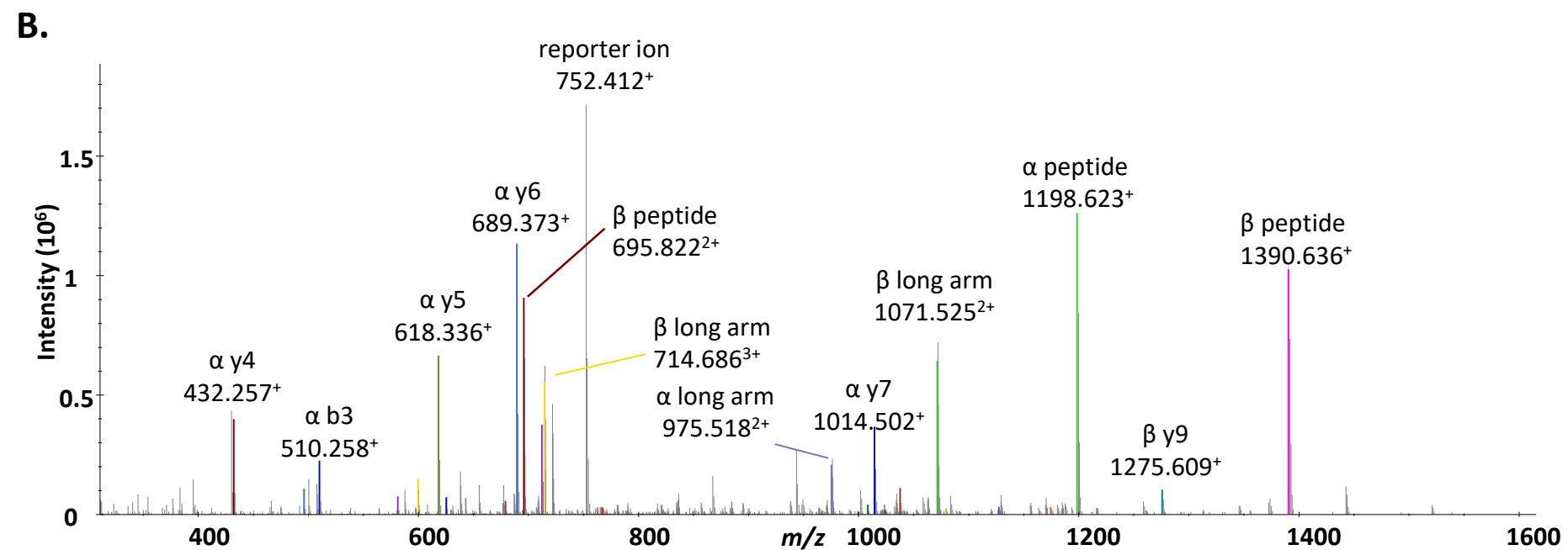

Supplement: S2 Fig — A. Schematic illustrating the PIR specific fragment ions generated upon collision induced dissociation of a cross-linked peptide pair linking K235 and K28 of BSA. Dissociation of the MS labile, aspartyl-prolyl peptide bonds in the PIR molecule result in the formation of released intact peptide ions for both the alpha and beta peptides which contain a residual mass modification (197.032 Da) referred to as a “stump mass” on the side chain of the cross-linked Lys. Dissociation of a single MS labile bond also results in the formation fragment ions containing a residual mass modification of (948.444 Da) on either the alpha and or beta peptides, referred to “long arm” ions. The reporter ion generated from dissociation of both MS labile bonds is observed at 752.412 m/z but is not generally used as a transition in PRM due to the non-specific nature of it being formed from dissociation of all PIR containing ions. B. MS2 spectrum for the cross-linked peptide pair mentioned in A with major fragment ions annotated. (PDF) [file pone.0167547.s002.pdf]

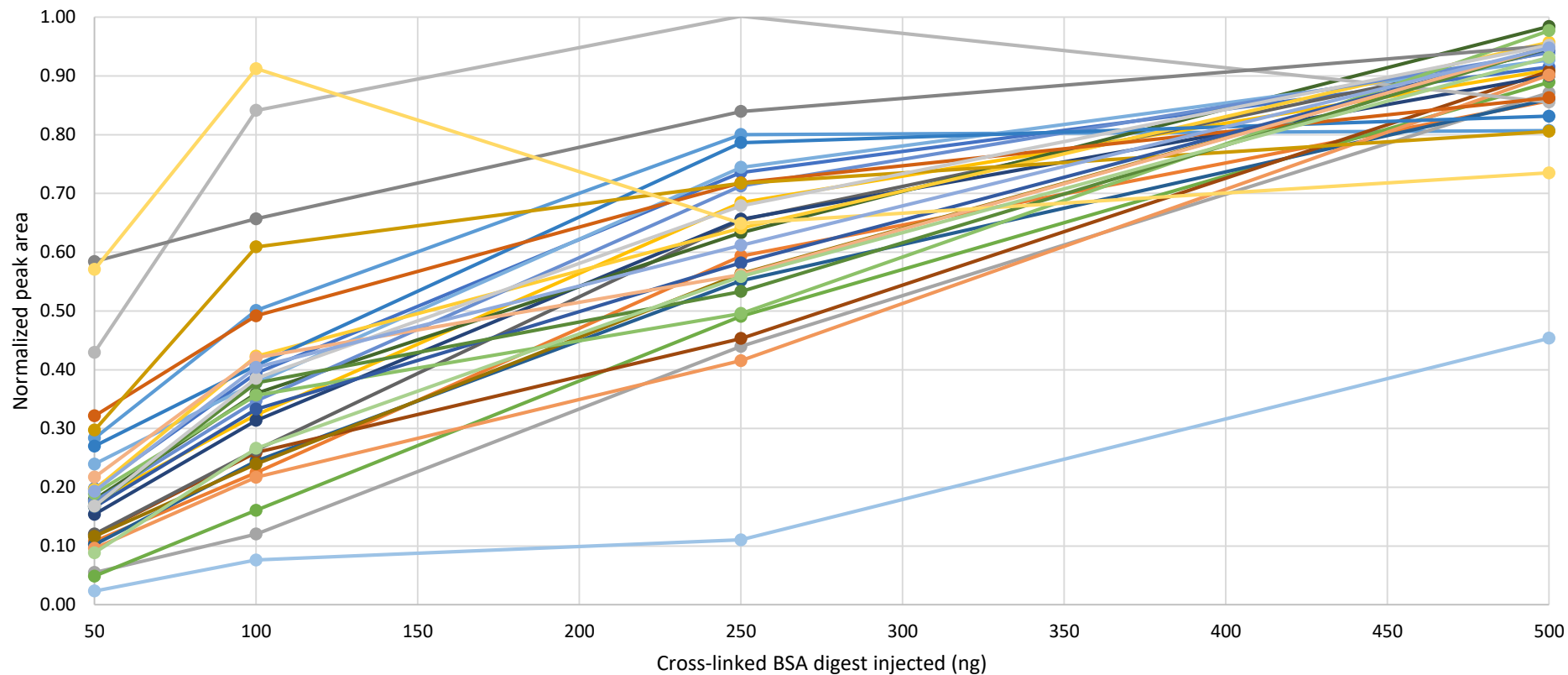

Supplement: S5 Fig — (PDF) [file pone.0167547.s005.pdf]
