# Supplementary material for: A General Method for Targeted Quantitative Cross-Linking Mass Spectrometry
Source: PLoS One. 2016 Dec 20;11(12):e0167547. doi: 10.1371/journal.pone.0167547 (PMC5172568; doi:10.1371/journal.pone.0167547)
Supplement: S3 Fig — Crystal structure of BSA (PDB 3V03) displayed as a ribbon structure with cross-linked Lys sites shown as green space filled models. The distances, in angstroms, between alpha carbon atoms of cross-linked Lys residues are shown as black labels. Note the residue numbering of PDB 3V03 differs from the BSA sequence numbering (UniProt entry ALBU_BOVIN) by -24 (i.e. K4 on structure is K28 in sequence). NGL viewer was used to create this image[28]. (PDF) [file pone.0167547.s003.pdf]

BSA structure PDB 3V03

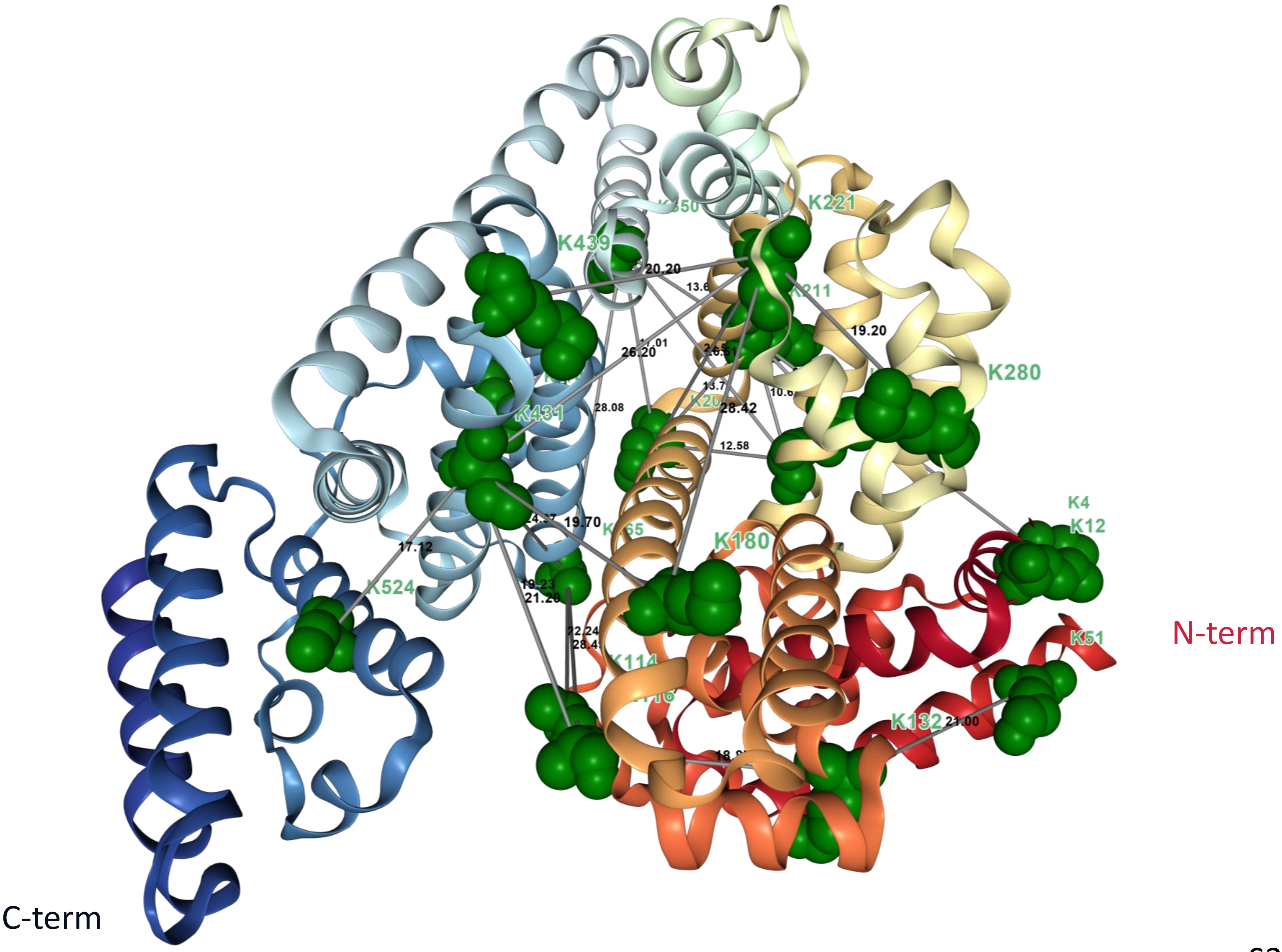

S3 Fig
