# Supplementary material for: A General Method for Targeted Quantitative Cross-Linking Mass Spectrometry
Source: PLoS One. 2016 Dec 20;11(12):e0167547. doi: 10.1371/journal.pone.0167547 (PMC5172568; doi:10.1371/journal.pone.0167547)
Supplement: S4 Fig — (PDF) [file pone.0167547.s004.pdf]

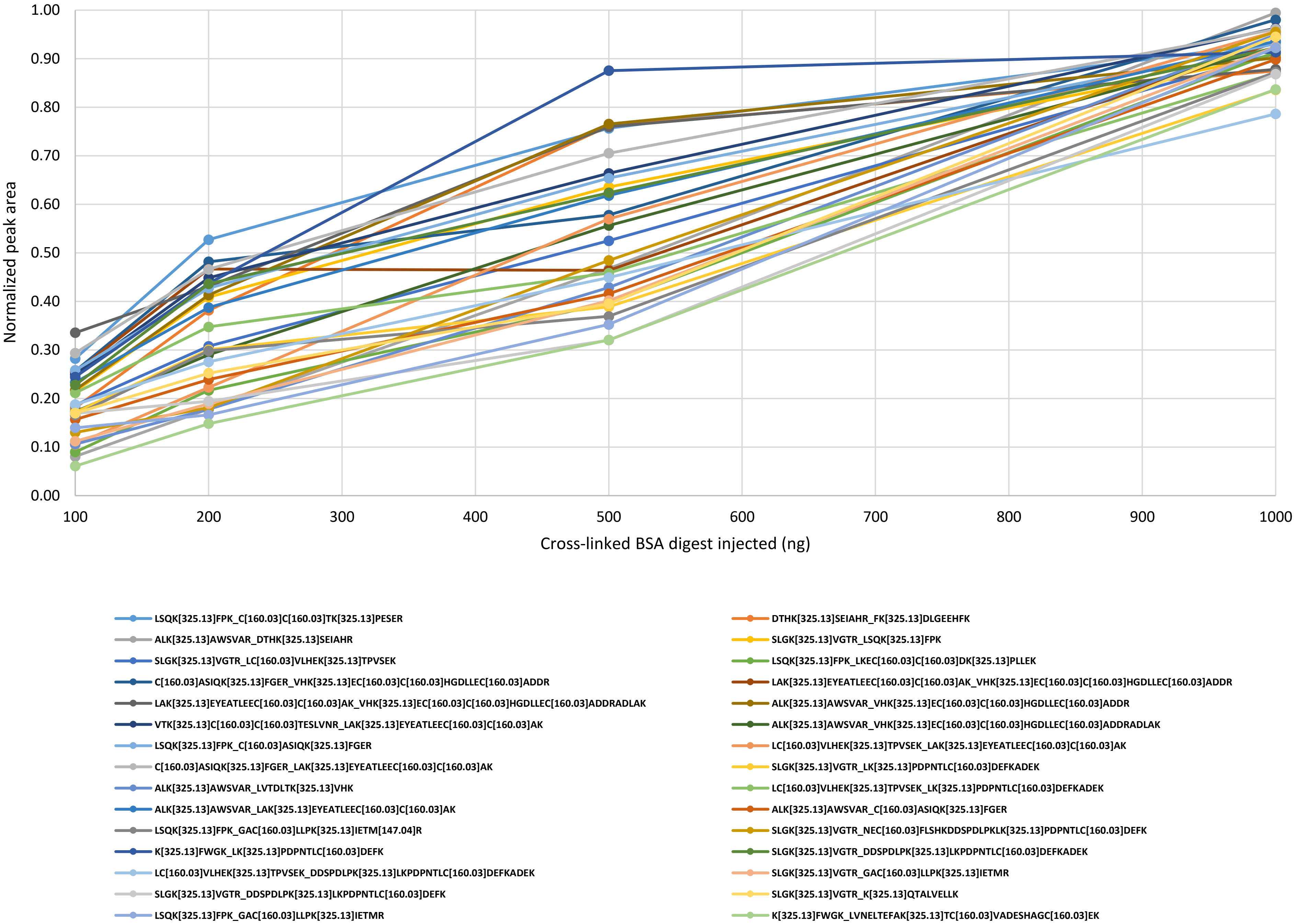

1.00
0.90
0.80
0.70
0.60
0.50
0.40
0.30
0.20
0.10
0.00
Normalized peak area
100
200
300
400
500
600
700
800
900
1000
Cross-linked BSA digest injected (ng)
LSQK[325.13]FPK_C[160.03]C[160.03]TK[325.13]PESER
ALK[325.13]AWSVAR_DTHK[325.13]SEIAHR
SLGK[325.13]VGTR_LC[160.03]VLHEK[325.13]TPVSEK
C[160.03]ASIQK[325.13]FGER_VHK[325.13]EC[160.03]C[160.03]HGDLLEC[160.03]ADDR
LAK[325.13]EYEATLEEC[160.03]C[160.03]AK_VHK[325.13]EC[160.03]C[160.03]HGDLLEC[160.03]ADDRADLAK
VTK[325.13]C[160.03]C[160.03]TESLVNR_LAK[325.13]EYEATLEEC[160.03]C[160.03]AK
LSQK[325.13]FPK_C[160.03]ASIQK[325.13]FGER
C[160.03]ASIQK[325.13]FGER_LAK[325.13]EYEATLEEC[160.03]C[160.03]AK
ALK[325.13]AWSVAR_LVTDLTK[325.13]VHK
ALK[325.13]AWSVAR_LAK[325.13]EYEATLEEC[160.03]C[160.03]AK
LSQK[325.13]FPK_GAC[160.03]LLPK[325.13]IETM[147.04]R
K[325.13]FWGK_LK[325.13]PDPNTLC[160.03]DEFK
LC[160.03]VLHEK[325.13]TPVSEK_DDSPDLPK[325.13]LKPDPNTLC[160.03]DEFKADEK
SLGK[325.13]VGTR_DDSPDLPK[325.13]LKPDPNTLC[160.03]DEFK
LSQK[325.13]FPK_GAC[160.03]LLPK[325.13]IETMR
DTHK[325.13]SEIAHR_FK[325.13]DLGEEHFK
SLGK[325.13]VGTR_LSQK[325.13]FPK
LSQK[325.13]FPK_LKEC[160.03]C[160.03]DK[325.13]PLLEK
LAK[325.13]EYEATLEEC[160.03]C[160.03]AK_VHK[325.13]EC[160.03]C[160.03]HGDLLEC[160.03]ADDR
ALK[325.13]AWSVAR_VHK[325.13]EC[160.03]C[160.03]HGDLLEC[160.03]ADDR
ALK[325.13]AWSVAR_VHK[325.13]EC[160.03]C[160.03]HGDLLEC[160.03]ADDRADLAK
LC[160.03]VLHEK[325.13]TPVSEK_LAK[325.13]EYEATLEEC[160.03]C[160.03]AK
SLGK[325.13]VGTR_LK[325.13]PDPNTLC[160.03]DEFKADEK
LC[160.03]VLHEK[325.13]TPVSEK_LK[325.13]PDPNTLC[160.03]DEFKADEK
ALK[325.13]AWSVAR_C[160.03]ASIQK[325.13]FGER
SLGK[325.13]VGTR_NEC[160.03]FLSHKDDSPDLPKLK[325.13]PDPNTLC[160.03]DEFK
SLGK[325.13]VGTR_DDSPDLPK[325.13]LKPDPNTLC[160.03]DEFKADEK
SLGK[325.13]VGTR_GAC[160.03]LLPK[325.13]IETMR
SLGK[325.13]VGTR_K[325.13]QTALVELLK
K[325.13]FWGK_LVNELTEFAK[325.13]TC[160.03]VADESHAGC[160.03]EK

S4 Fig
