# Supplementary material for: A General Method for Targeted Quantitative Cross-Linking Mass Spectrometry
Source: PLoS One. 2016 Dec 20;11(12):e0167547. doi: 10.1371/journal.pone.0167547 (PMC5172568; doi:10.1371/journal.pone.0167547)
Supplement: S6 Fig — Entry form for PRM transition calculator can be obtained at the following URL: http://xlinkdb.gs.washington.edu/xlinkdb/prmTransitionForm.php. (PDF) [file pone.0167547.s006.pdf]

Generate a PRM transition .txt file for analysis of cross-links

PRM Transition Information:

Peptide A: FYEAFSKNLK

Peptide B: FYEQFSKNIK

Site A: 7

Site B: 7

Cross-linker Type:

- (•) BDP-NHP
- ( ) DSSO
- ( ) Other

Precursor Charge State:

- (•) 4+
- ( ) 5+
- ( ) 6+

Modifications:

Peptide A:

- ( ) None.
- (•) use carbamidomethyl cysteine (C, monoisotopic) - 57.021464
- ( ) User Defined Modifications:

#@pos, i.e. "16.0@2 57.1@3", space separated for addition to N-term use '[' and for C-term use ']', e.g. "16.0@["

Peptide B:

- ( ) None.
- (•) use carbamidomethyl cysteine (C, monoisotopic) - 57.021464
- ( ) User Defined Modifications:

#@pos, i.e. "16.0@2 57.1@3", space separated for addition to N-term use '[' and for C-term use ']', e.g. "16.0@["

Generate PRM Transitions

S6 Fig
