# Supplementary material for: A General Method for Targeted Quantitative Cross-Linking Mass Spectrometry
Source: PLoS One. 2016 Dec 20;11(12):e0167547. doi: 10.1371/journal.pone.0167547 (PMC5172568; doi:10.1371/journal.pone.0167547)

Text S1. Protocol for using Skyline for cross-linked peptides

1. Open Skyline and create a blank document. Open the transition list:

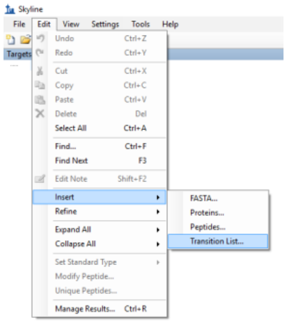

2. Select the small molecules option and set up the columns in the transition list table in the following order:

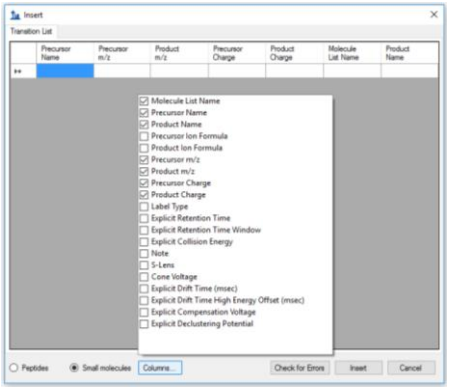

3. Open the tab-delimited text file output from ReACT2prm with a text editor (we recommend Notepad++, https://notepad-plus-plus.org/) and copy paste the information into the Skyline transition list table then click check for errors:

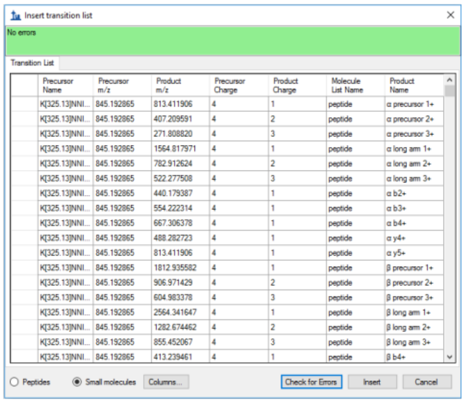

4. Click insert and the transitions for the cross-linked peptide pairs will be uploaded into Skyline:

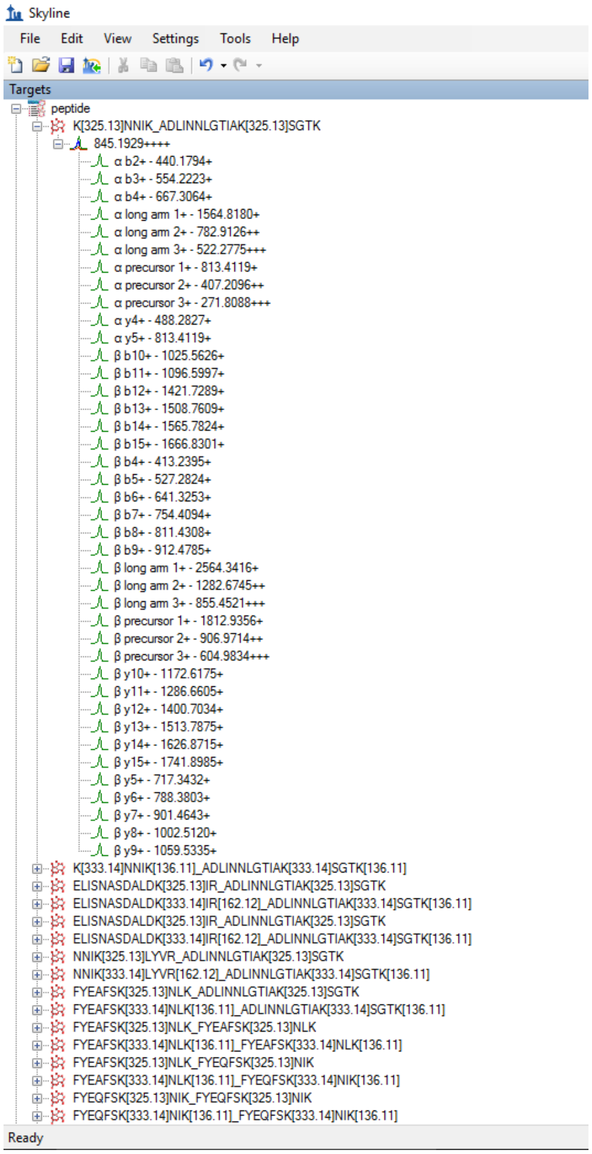

5. To export an isolation list for building a PRM method click file->export->isolation list, select the instrument type to be used and click OK:

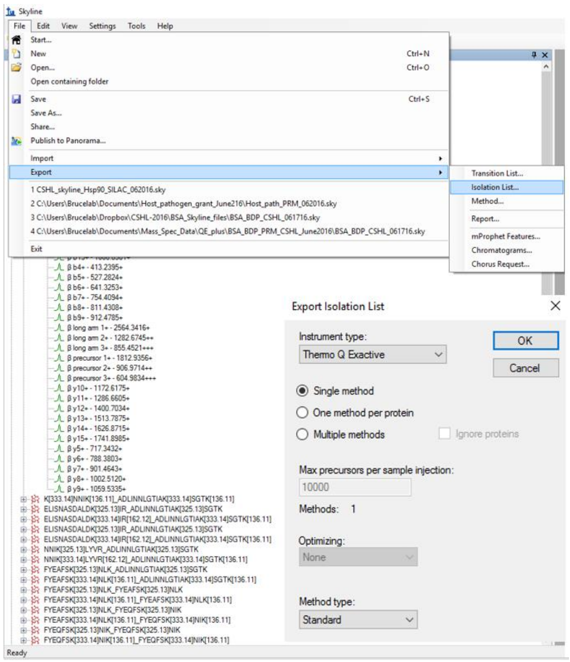

6. At this point the Skyline document can be saved. After collection of PRM mass spectral data Skyline can be used to analyze the data by importing the raw files:

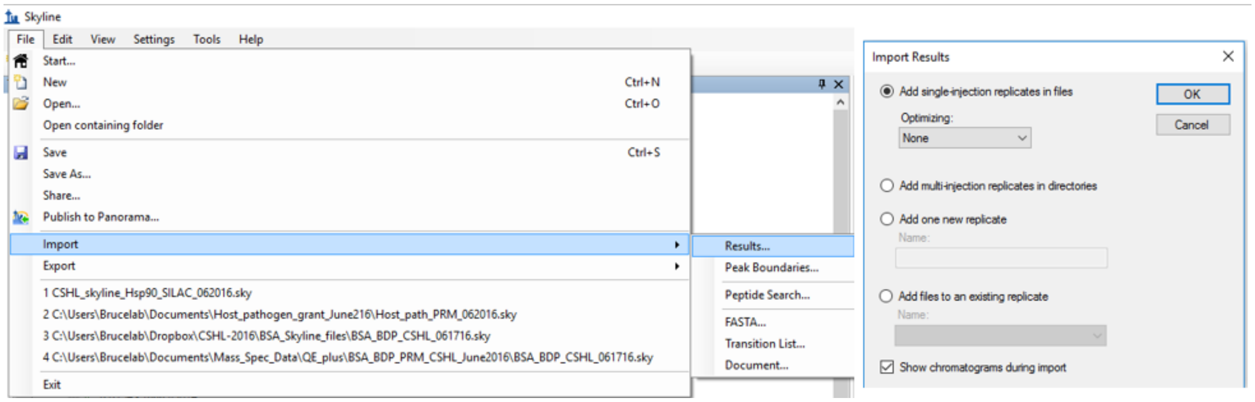

Supplement: S1 Text — (PDF) [file pone.0167547.s008.pdf]
